# Emerging Issues and Initial Insights into Bacterial Biofilms: From Orthopedic Infection to Metabolomics

**DOI:** 10.3390/antibiotics13020184

**Published:** 2024-02-13

**Authors:** Rasoul Mirzaei, Davide Campoccia, Stefano Ravaioli, Carla Renata Arciola

**Affiliations:** 1 Venom and Biotherapeutics Molecules Laboratory, Medical Biotechnology Department, Biotechnology Research Center, Pasteur Institute of Iran, Tehran 1316943551, Iran; rasul.micro92@gmail.com; 2 Laboratorio di Patologia delle Infezioni Associate all’Impianto, IRCCS Istituto Ortopedico Rizzoli, Via di Barbiano 1/10, 40136 Bologna, Italy; davide.campoccia@ior.it (D.C.); stefano.ravaioli@ior.it (S.R.); 3 Laboratory of Immunorheumatology and Tissue Regeneration, Laboratory of Pathology of Implant Infections, IRCCS Istituto Ortopedico Rizzoli, Via di Barbiano 1/10, 40136 Bologna, Italy; 4 Department of Medical and Surgical Sciences (DIMEC), University of Bologna, Via San Giacomo 14, 40126 Bologna, Italy

**Keywords:** biofilm, orthopedic implant infections, canaliculo-lacunar network reservoir, bacterial dormancy, immunometabolism, internalization, metabolomics

## Abstract

Bacterial biofilms, enigmatic communities of microorganisms enclosed in an extracellular matrix, still represent an open challenge in many clinical contexts, including orthopedics, where biofilm-associated bone and joint infections remain the main cause of implant failure. This study explores the scenario of biofilm infections, with a focus on those related to orthopedic implants, highlighting recently emerged substantial aspects of the pathogenesis and their potential repercussions on the clinic, as well as the progress and gaps that still exist in the diagnostics and management of these infections. The classic mechanisms through which biofilms form and the more recently proposed new ones are depicted. The ways in which bacteria hide, become impenetrable to antibiotics, and evade the immune defenses, creating reservoirs of bacteria difficult to detect and reach, are delineated, such as bacterial dormancy within biofilms, entry into host cells, and penetration into bone canaliculi. New findings on biofilm formation with host components are presented. The article also delves into the emerging and critical concept of immunometabolism, a key function of immune cells that biofilm interferes with. The growing potential of biofilm metabolomics in the diagnosis and therapy of biofilm infections is highlighted, referring to the latest research.

## 1. Introduction

Our understanding of bacterial biofilm has improved since it was identified in the last century. Research advances are providing new details about biofilm, illuminating its different facets, and will help to better understand the nature of microbial biofilms. 

From a broad perspective, biofilms predominate in all habitats on the Earth’s surface, with 40–80% of prokaryotic cells on Earth residing in biofilms [1]. Most cells organized as biofilms are found beneath continents and under the ocean floor (Flemming and Wuertz 2019). It has been proposed that biofilms drive all biogeochemical processes and represent the major form of active bacterial life [1]. 

In the clinical field, the hope—or rather, the expectation—is that advances will allow us to improve diagnosis and therapy and, therefore, to fight biofilm infections more and more effectively. 

Importantly, biofilms are recalcitrant to medical treatments due to their ability to resist or tolerate antibiotics and evade immune defenses, which hinders the treatment of infections [2]. In orthopedics, the management of implant-associated biofilm infections is one of the most complex, requiring a multidisciplinary-team approach, including orthopedic surgeons, radiologists and medical imaging specialists, microbiologists, infectious disease specialists, physicians, pathologists, plastic surgeons, physiatrists, and basic scientists [3]. 

Implant-associated infection affects approximately 5% of elective and emergency orthopedic surgery procedures, representing a significant surgical complication, which often leads to implant failure [4]. Additional subsequent surgeries are often required, characterized by high morbidity rates and significant financial burdens. In some cases, these interventions can culminate in the unfortunate outcomes of amputation or death [5,6].

According to the classic model of implant infection, once the bacteria have successfully attached themselves to the implant, the insidious and threatening bacterial consortium known as biofilm forms [7]. 

Biofilm-related implant infections represent a significant burden on healthcare systems, comprising 65% of all bacterial infections in healthcare facilities [8].

Biofilm does not lend itself to a simple and limited description. Cells embedded in biofilms exhibit altered metabolism and growth rate, as well as different gene expression, compared to planktonic cells [9]. The diversity of microorganisms living in biofilms and the complex nature of the biofilm matrix at the pathogen–host interface contribute to the difficulties in studying biofilm. On the other hand, it is now known that the production of biofilms exacerbates the infection, making it chronic. Thus, biofilm-associated infections include chronic urinary tract infections (UTIs), recurrent tonsillitis, rhinosinusitis, otitis media, periodontitis, cystic fibrosis, device-related infections (such as catheter-related infections), wound infections, and orthopedic implant-related infections [10].

Although much research has been successfully conducted and considerable information has been obtained about biofilms, some key questions remain unanswered. Given the implications for future treatments of chronic biofilm-associated infections, gaining further knowledge is critical. This is especially true for biofilm infections associated with orthopedic implants, which often cause any treatment to fail. Overall, biofilms have been studied in recent years, and there is continuous progress. 

In this review, we comment on the latest developments and discoveries on biofilms, also reflecting on the questions that are still open and still waiting to be answered. This paper focuses on biofilms in orthopedic implants, examining the issues, needs, pathogenetic knowledge, and innovations useful for diagnosis and therapy. Special attention is paid to examining the implications and opportunities generated by the evolution of knowledge on the mechanisms of bacterial persistence associated with biofilm formation, on the crosstalk between host cells and biofilm, and on the relevant aspects that emerge from the study of immunometabolism.

## 2. From Ancient Times until Today: A Brief Historical Excursus on Biofilm

To date, research and the understanding of bacterial biofilm has made significant progress, and the field continues to evolve in several key areas. The first microscopic observation of biofilm was by Anthony van Leeuwenhoek in the 17th century [11]. He was the first to observe, with a microscope he designed himself, the “animalcules” on the surface of his teeth, realizing that, even after cleaning, they remained “as thick as if it were batter” [11]. 

Biofilms were not recognized and defined until 1978. In 1978, biofilm research pioneer John William (Bill) Costerton launched an entirely new microbiological theory called biofilm theory. The basis of the new theory was the concept that bacteria form consortia to face adversity and hostile environments by coming together and encapsulating themselves in a protective matrix [12,13]. Bill Costerton was a pioneer in the study of biofilm in the areas of physiology, biochemistry, biomaterials, and so on [10,13]. 

At the same time, Niels Høiby, a medical microbiologist at the University of Copenhagen, began research on cystic fibrosis [14]. He observed bacterial aggregates through the microscopic examination of sputum from patients with chronic lung infection by *Pseudomonas aeruginosa* [14].

In May 2019, four decades after Costerton’s official discovery of biofilm and seven years after his death [12,13], a meeting was held in Leavenworth, USA, aimed at identifying gaps in the knowledge of biofilm and ways to bridge them [15]. The basic idea was to holistically study biofilms in a variety of clinical and non-clinical fields, such as osteomyelitis, medical device infections, native valve endocarditis, cystic fibrosis, and dental plaque, among clinical fields, but also metal corrosion, oil degradation, and others, among the non-clinical ones [15]. Two of the prominent clinical findings that emerged from that meeting are indicated hereafter: the need to search for new non-antibiotic or antibiotic adjuvant prevention and treatment strategies, since surgical curettages and conventional antibiotics fail to treat biofilms; and the lack of comprehensive imaging methods to detect biofilms, which makes it difficult to evaluate the effectiveness of new antibiofilm treatments. 

As far as this latter point is concerned, there is no doubt that the future of biofilm research will be critically founded on the development and application of new (imaging) technologies for clinical diagnostics.

Current clinical biofilm research is also exploring in depth the role of biofilms in various chronic diseases. These pathogenetic studies are aimed at better understanding the complex microbial interactions within biofilms and between biofilms and microenvironments.

## 3. How Staphylococci Escape Host Defenses and Antibiotics in Peri-Implant Infections

Staphylococci are the main cause of implant-associated infections in orthopedics [16,17]. Thus, a detailed knowledge of the key pathogenetic mechanisms leading to their success is of fundamental importance. 

Biofilm formation, dormancy within biofilm, internalization into osteoblasts, and penetration into cortical bone canaliculi are strategies used by staphylococci to survive at the implant–periprosthetic bone interface, eluding host immunity and resisting antibiotics [2,10].

**Figure 1:**
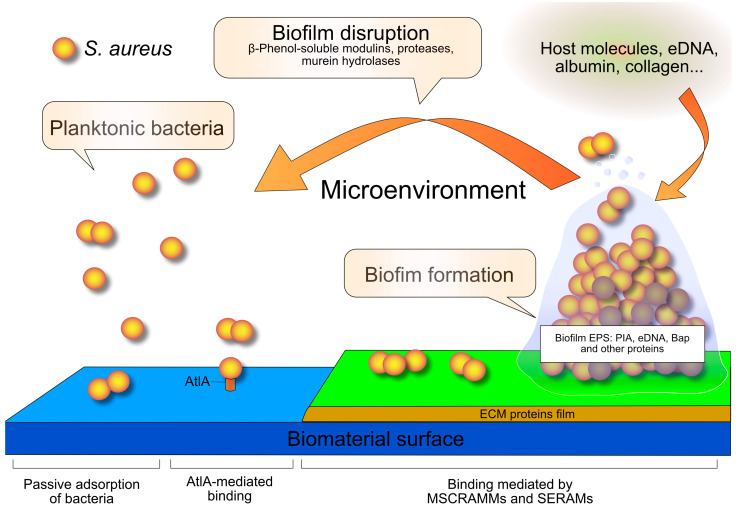
The classical model of the biofilm cycle: the four-step process of biofilm formation in *S. aureus.* Planktonic cells adhere to the biomaterial surface through AtlA adhesin. Then, MSCRAMMs and SERAMs bacterial adhesins interact with the extracellular matrix (ECM) proteins coating the implant. Bacteria proliferate and produce an extracellular polymeric substance consisting of proteins (among which is the biofilm-associated protein Bap), the intercellular polysaccharide adhesin (PIA), and a series of other polymeric extracellular substances, among which is extracellular DNA (eDNA). Once a mature biofilm has formed, under the control of the quorum sensing system, the enzymes β-phenol-soluble modulins, proteases, and murein hydrolases dissolve biofilm and release planktonic bacteria to initiate a new biofilm cycle. The more recently emerged role of the microenvironment and host molecules in biofilm formation is also recalled. Abbreviations: AtlA, the major autolysin of *S. aureus*; EPS, extracellular polymeric substance; MSCRAMMs, Microbial Surface Components Recognizing Adhesive Matrix Molecules; SERAMs, Secretable Expanded Repertoire Adhesive Molecules.

According to the classical model, biofilm formation on an orthopedic implant is a four-step process [10] (see Figure 1). In the initial step, bacteria are passively adsorbed, as they are driven by hydrophobic, electrostatic, and Van der Waals forces. In addition, the major autolysin of *Staphylococcus aureus* (AtlA) and of *Staphylococcus epidermidis* (AtlE) mediate the binding to the abiotic surfaces [10,18,19,20]. Autolysins also mediate staphylococcal internalization [21,22].

Specific adhesins bridge to the host matrix proteins that cover the implant surface. In *S. aureus*, adhesins comprise the cell wall-anchored Microbial Surface Components Recognizing Adhesive Matrix Molecules (MSCRAMMs [23]) and the Secretable Expanded Repertoire Adhesive Molecules (SERAMs) [24]. In *S. aureus* from orthopedic implant-infections, the *bbp* (for “bone sialoprotein-binding protein”) and *cna* (for collagen adhesin) couple of genes appeared to be an important virulence trait [25,26], which could favor or determine their success as implant colonizers.

The extracellular polymeric substance (EPS) of the biofilm is mainly composed of polysaccharides, proteins, teichoic acids, and extracellular DNA. [10,27]. The principal polysaccharide is the Polysaccharide Intercellular Adhesin (PIA), a linear homoglycan composed of β-1,6-linked *N*-acetylglucosamine residues. PIA synthesis is encoded by the intercellular adhesion (*icaADBC*) locus [18]. This locus was first recognized in *S. epidermidis* [28], then in *S. aureus* [29,30]. In the bacterial genome of *S. epidermidis*, no single *ica* genes were found to be missing, as either all genes were present or the entire locus was absent [31]. 

Importantly, PIA-producing strains exhibit high resistance and polyresistance to antibiotics, mainly to aminoglycosides, sulfamethoxazole, and ciprofloxacin [32]. 

The mobile element, IS256, previously related to the phase variation of biofilm formation in *S. epidermidis* [33] and then to multi-antibiotic resistance [34], has more recently been associated with bacterial genome plasticity in adapting to antibiotic stress [35,36]. In a very recent study, Kirsch et al. showed that stressors, such as antibiotic exposure, drive genome transposition in enterococci, suggesting that the diversification of IS256 can explain how selective pressure mediates the evolution of the enterococcal genome and the emergence of dominant nosocomial lineages [37].

The role of polysaccharide intercellular adhesin in biofilm along with its structural and regulatory aspects are reviewed in [38]. Biofilm production can also follow *ica*-independent pathways [39]. These rely on the expression of cell wall adhesins that maintain a cell-to-cell interaction inside the biofilm. The first recognized was the biofilm-associated protein Bap [40]. In clinical isolates of *S. aureus* and *S. epidermidis* from total hip and knee infected arthroplasties, over a quarter of *S. epidermidis* biofilm-producing strains were PIA-independent and expressed the accumulation-associated protein Aap [41]. In *S. aureus*, SasC and SasG (homologues of the *S. epidermidis* Aap), the clumping factor B (ClfB), the serine-aspartate repeat protein SdrC, the protein A, the fibronectin/fibrinogen-binding proteins FnBPA and FnBPB, also promote the accumulation of biofilm [23,41,42]. 

Another EPS component is extracellular DNA (eDNA). eDNA production proceeds as follows. In *S. aureus*, some cells kill themselves through programmed cell death [43] for the benefit of the microbial community. Differently, in *E. faecalis*, killer cells suppress victim cells [44]. In both cases, eDNA comes from dead cells. In *S. epidermidis*, eDNA release is mediated by AtlE [45]. eDNA strengthens the biofilm, acts as a gene pool for horizontal gene transfer, serves as a source of nutrients, and prevents phagocytosis [43,46,47]. Moreover, eDNA contributes to the complex architecture of the bacterial biofilm matrix through a variety of interactions with other molecular components of the biofilm matrix [27,46].

Staphylococci escape host defenses and antibiotics not only by wrapping in biofilms, but also by hiding in eukaryotic cells, impermeable to conventional extracellular antibiotics. In this regard, FnBPA and FnBPB *S. aureus* adhesins are essential for bacterial invasion in eukaryotic cells [48], although FnBPA plays a more crucial role than FnBPB [49].

**Figure 2:**
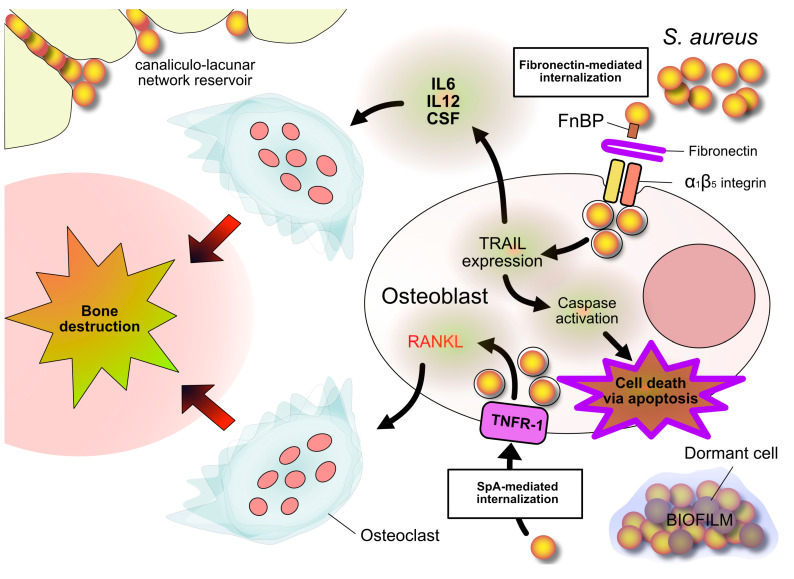
*S. aureus* internalization into osteoblasts. Two entry mechanisms are depicted. One is mediated by fibronectin, which forms a bridge between staphylococcal FnBP adhesin and osteoblast α_1_β_5_ integrin. This interaction promotes expression of Tumor Necrosis Factor-Related Apoptosis Inducing Ligand (TRAIL), which induces caspase 8 activation and the consequent osteoblast apoptosis. The other way is mediated by the interaction of Staphylococcal protein A (SpA) and Tumor Necrosis Factor Receptor 1 (TNFR-1) on the osteoblast surface. This interaction promotes the expression of the Receptor Activator of NF Kappa B Ligand (RANKL), a key cytokine in promoting osteoclastogenesis. Bone destruction results from the apoptotic death of osteoblasts and from the activation of osteoclasts. Other, cytokines released by internalized osteoblasts recruit monocytes/macrophages and induce their differentiation into osteoclasts, thus corroborating osteoclastogenesis. Also depicted are bacteria nestled in the bone canaliculi and dormant bacteria within biofilm.

The internalization of *S. aureus* into osteoblasts has been ascribed to the mechanisms depicted in Figure 2. *S. aureus* and *S. pseudintermedius* turned out to be competent, while *S. epidermidis*, *S. lugdunensis,* and *E. faecalis* turned out to be incompetent, to enter osteoblasts [50,51].

Interestingly, studies have shown that bacteria can penetrate the bone canaliculi, thus continuing their proliferation while remaining out of sight and out of reach of the host’s immune cells [52,53]. Those who study biofilms know how much bacteria love niches. Yet the observation that bacteria colonize canaliculi is relatively recent. Morphological insights into canaliculi are also recent. The porous network that houses the osteocyte system is called the bone lacuno-canalicular network. Here, osteocytes transfer nutrients, biochemical signals, and hormonal stimuli that allow for their interaction with other bone cells. The canaliculi are the branches of this system. A 2020 study is dedicated to the assessment of the human bone lacuno-canalicular network at a nanoscale, and another just-published 2023 study concerns the imaging of bone disease through the near-infrared-II window [54,55]. Canaliculi have an average diameter of approximately 100–600 nm (0.1–0.6 µm); therefore, they have a diameter smaller than that of *S. aureus* (1 µm) and approximately corresponding to that of *S. epidermidis* (0.5 µm), the two main etiological agents of orthopedic implant infections. This means that bacteria cannot enter the canaliculi easily. However, some pathological conditions, such as osteoporosis, can widen the diameter of bone canaliculi.

We would like to draw attention to the fact that one of the problems that make the complete eradication of the biofilm difficult is the presence of metabolically dormant “persister” cells, against which common antibiotics or metabolic antibacterials may not be effective [56]. The first observation of persistent bacterial cells is attributable to Joseph Bigger who, way back in 1944, observed that among staphylococci exposed to intermittent sterilization with penicillin some bacterial cells survived [57]. In implant-related infections, following treatment with antibiotics, a small part of the bacterial population becomes dormant. Persistent cells do not grow but survive, and can reactivate, multiply, and reconstitute a biofilm when antibiotic therapy ceases [58]. Sub-lethal concentrations of antibiotics and stresses favor the phenomenon of bacterial dormancy [59,60]. Killing dormant cells cannot be achieved with traditional antibiotics, which require active metabolism by the affected bacterial cells. Researchers’ attention is turning to antimicrobial peptides (AMPs), whose bactericidal effect lies in the ability to damage the bacterial membrane. Some AMPs have already been approved for clinical use, while others are currently facing advanced clinical trial phases. Therefore, new AMPs are compounds to be searched for and tested as antibacterial/antibiofilm candidates [61]. 

Contact between AMPs and the bacterial membrane should also be encouraged, as it can be hindered by the biofilm matrix. New compounds that are capable not only of killing bacteria but also of reaching them deep in the biofilm is a desirable solution. A recent article by authors from Nanjing, China, reports of a conjugate between AMP and a furoxan moiety, which seems to produce *S. aureus* and *Escherichia coli* biofilm eradication and dispersion [62]. This methodology could be conveniently adapted and applied to prefigure new compounds containing different antibiofilm/dispersing agents. However, these approaches still require long periods of time to be translated to a clinical use.

## 4. Alternative Models of Biofilm Formation

The classic model here recalled explains biofilm formation and its propagation in a simple way. However, the latest studies question this classic model, underlining some critical aspects: (i) the classical model refers to in vitro conditions, and it does not consider the importance and variety of the in vivo microenvironment [63]; (ii) in the presence of an implant, biofilms can form either by adhering to the surface of the implant or by interacting with the periprosthetic tissues, not necessarily using both ways in every infection [64]; and (iii) the classical model does not consider the non-attached biofilms [65]. 

We discuss these three points concisely below.

Very recent articles have focused on the importance of understanding the infectious microenvironment (see also the next paragraph). This, in fact, could appreciably influence bacterial behaviors and the susceptibility of the biofilm to treatments. The infectious microenvironment could cause sensitive bacteria to lose their susceptibility to antibiotics that are effective in standard laboratory susceptibility testing. In this regard, it is a common clinical observation that standard doses of antibiotics do not efficiently treat chronic infections of the soft tissue and bone. The behaviors that bacteria exhibit under standard laboratory conditions can be substantially different from those they adopt when infecting living tissues. The infectious microenvironment could lead to changes in bacterial metabolism that result in increased protection against antibiotics. Therefore, it can be expected that antibiotic treatment will be more effective when antibiotics are chosen based on in vitro susceptibility tests in which the real infectious microenvironment is recreated. In this connection, proteins’ adsorptions on titanium and bone samples were found to be crucial factors that influence *S. aureus* biofilm formation, the difference in materials reverberating not only on adhesion, but also on the matrix composition and biofilm-related gene regulation. These results highlight the need for new models for the study of biofilms that are representative of in situ conditions. This will allow us to better evaluate and prefigure therapeutic strategies against biofilms.

The second point of attention is the following. It is stated that bacteria adhere to the surface of the biomaterial, as can be deduced from the classic biofilm model. However, recent studies suggest that, although bacteria in periprosthetic infections can adhere to the surface of the biomaterial, they can also be located only in the periprosthetic tissues, and not necessarily on the implant. Noticeably, detached aggregates exhibit the same antibiotic tolerance observed in surface-adherent biofilms [64]. 

If the possibility that bacteria colonize the periprosthetic tissues—without necessarily adhering to the implant material—is established, research on anti-adhesive or contact-killing coatings [66,67] (biomaterial technologies both inspired by the classical model in which adhesion to the surface of the biomaterial is the primum movens of infection) should be revisited, emphasizing the design of biomaterials that release drugs to reach the bacteria nested in the tissues [68]. 

We add that there is an urgent need for research into new molecules that are alternatives to antibiotics, effective, and non-toxic, which do not induce drug resistance, and which are also effective on intracellular bacteria, on dormant bacteria, and on bacteria hidden in the depths of the bone canaliculi: this is a great challenge.

The third critical point concerns the so-called non-attached biofilm aggregates, which consist of bacteria submerged into an extracellular matrix. A very recent review presents and discusses the characteristics of non-attached biofilm aggregates [65]. In the article, the following aspects are examined: the mechanisms by which they form and disperse; the in vitro models for their analysis; several examples of their occurrence; their tolerance to antibiotics and the evasion of immune responses; and a comparison between non-attached biofilm aggregates and surface-attached biofilms. 

Bjarnsholt et al. defined in a meta-analysis the distribution of biofilm aggregates in chronic infections. They reported the presence of non-attached aggregates in cystic fibrosis, chronic wounds, otitis media, and chronic osteomyelitis [65,69]. Interestingly, many of these non-attached biofilms are small bacterial aggregates surrounded by polymers-rich host secretions and large numbers of inflammatory cells [65,70]. Domnin et al. proposed an in vitro model of non-attached biofilm-like bacterial aggregates, based on magnetic levitation, to study non-attached aggregates with confocal laser scanning microscopy (CLSM) and scanning electron microscopy (SEM), and to characterize them quantitatively. Using this model, they demonstrated that, despite morphological and functional similarities between non-attached aggregates and biofilms, biofilm-forming strains can exhibit poor non-attached aggregate formation, suggesting that the mechanisms underlying the formation of adhered biofilms differ from those leading to non-attached aggregates [65].

## 5. Can the Biofilm Formation Be Induced by Host-Derived Factors?

When a biofilm forms, bacteria change from their planktonic state to an aggregated state that is peculiarized by a blanket of extracellular polymeric substances (EPSs), the so-called biofilm matrix. When exposed to a variety of micronutrients, bacterial cells produce enzymes that change and modulate the composition of EPSs [71,72].

Further studies are needed to fully define the complex interactions between hosts and bacterial biofilms. When possible—which occurs when the bacteria are in the planktonic phase or are in the very initial phase of the formation of their biofilm—the host expresses a highly effective defense against bacterial invaders. Bacteria, for their part, use programmed feedback and skillful strategies of defense, evasion, and even the hijacking of host defenses to pursue their advantage and safeguard their survival. Sometimes, host immune responses are insufficient or ineffective, which allows bacteria to create robust biofilms with thick matrices to shield themselves from host defenses. 

It is hypothesized that bacterial biofilm production may include in the matrix either a layer of molecular components released by host cells or an incorporation of them within the matrix. These host molecules would enrich the components produced by the bacteria themselves. For example, host-specific eDNA may play a key role in the formation of bacterial biofilms in the lungs of cystic fibrosis patients [8,73]. The interaction between eDNA and Psl (a *P. aeruginosa* exopolysaccharide) serves to enrich the biofilm scaffold and favors the survival of *P. aeruginosa* during lung colonization. 

Gallo et al. demonstrated that salmon spermatozoa-derived eukaryotic DNA molecules could be absorbed into bacterial amyloid fibers [74]. 

Among the components incorporated into the biofilm are, in addition to DNA, albumin, collagen, and mucin [75]. 

In addition, Walker et al. found that human neutrophil cells are mediators of *P. aeruginosa*’s increased biofilm production [76]. This correlates with the finding that, when the host immunity is unable to eliminate bacterial infections, host-derived components from necrotic immune and non-immune cells (cellular debris, biological molecules, inorganic nanomaterials) could serve as a welcome resource that encourages bacteria to enrich the matrices of their biofilms, by weaving them, or reweaving them, more sumptuously. 

Rhamnolipids are a class of glycolipid produced by *P. aeruginosa*. They express cytolytic activity toward host cells. Neutrophils in the proximity of the biofilm produce host-derived debris, including DNA and actin polymers, which *P. aeruginosa* exploits as an additional source of material for assembling and shaping the biofilm matrix [77]. Host debris has been identified in the sputum of cystic fibrosis patients. The disruption of these polymers breaks the *P. aeruginosa* biofilm and reduces the pace of biofilm development [77]. 

Another previously mentioned important aspect to recall is that most of the knowledge on biofilms still derives from in vitro studies. Indeed, while a great deal of information on the molecular factors involved in the in vitro generation of bacterial biofilm has been gathered, the definitions of biofilm formation processes within the host is considerably less clear. However, as already mentioned, host components are hypothesized to play a significant role in the formation of bacterial biofilms in vivo; consequently, in the clinic, these host components must be taken into consideration when evaluating the efficacy of new therapies to target bacterial biofilms.

Rahman et al. evaluated the viscoelasticity of in vivo *P. aeruginosa* biofilms through ex vivo micro-rheology measurements of in vivo biofilms excised from mouse wound beds. The in vivo results were compared to typical in vitro measurements. Biofilms grown in vivo are more elastic than those grown in vitro. It was observed that the contribution of exopolysaccharides to the viscoelasticity of *P. aeruginosa* biofilms was different between biofilms grown in vitro and biofilms grown in vivo. In vitro experiments with collagen-containing media suggested this phenomenon may be attributable to the incorporation of host material, most notably collagen, into the matrix of the in vivo-grown biofilms. Collagen appeared to be the dominant contributor to biofilm viscoelasticity in vivo [78]. 

Garcia-Bonillo et al. studied the role of human albumin in the formation of bacterial biofilms on urinary catheters by simulating environmental and physical conditions using a quartz crystal microbalance with dissipation. They demonstrated that human albumin can be considered a promoter of biofilm formation on hydrophobic surfaces, being a possible risk factor to developing catheter-associated urinary tract infections [79]. 

Wu et al. found that mucin shapes microbial communities in several ways: serving as a nutrient to support metabolic diversity, organizing spatial structure through reduced aggregation, and limiting antagonism between competing taxa. Overall, the work of Wu et al. identified mucin glycans as a natural host mechanism [80]. 

Skovdal et al. demonstrated that host plasma components cause changes in biofilm formation and composition in *S. epidermidis* [81]. They pointed out that some of the biofilm matrix components previously thought to be important for biofilm formation, such as PIA, do not appear as critical in the presence of host factors. They reasoned that the role of PIA in biofilms might be predominant in in vitro cultured biofilms, which form in sugar-rich laboratory media. Conclusively, they suggested that in vitro models used to study biofilm infections must include host factors in the growth media. 

Consistently, the study by Christner et al. demonstrated that PIA production is downregulated in human serum [82]. In *S. epidermidis*, the formation of a proteinaceous biofilm relies on the extracellular matrix binding protein Embp, a giant protein endowed in the cell wall of *S. epidermidis* that mediates the binding of *S. epidermidis* to (surface-attached) fibronectin [83]. In contrast to PIA, Embp is present in almost all clinical isolates, and it is upregulated when *S. epidermidis* grows in human serum, this suggesting an essential role of Embp. 

The microenvironment can also have a strong impact on antibiotic resistance.

An interesting study by Xin et al. explores a world—that of the sewers—apparently far from the clinic but important in a holistic vision of medicine such as that represented by the modern concept of One Health. The mentioned study highlighted that trace antibiotics favor the transfer of antibiotic resistance genes by regulating the extracellular polymeric substances of biofilm in sewers [84]. 

Townsley et al. reported that (natural-product) antibiotics at subinhibitory concentrations can impact biofilm formation in neighboring bacteria and hypothesized that these compounds mediate the biofilm formation and the cell–cell interactions [85]. 

Bernardi et al. showed that subinhibitory concentrations of the antibiotics tetracycline and doxycycline promote biofilm formation by *Enterococcus faecalis* [86]. We would like to take this opportunity to recall that *E. faecalis* is the fourth bacterial species in order of frequency to colonize periprosthetic tissues and that it is a formidable biofilm former.

In a recently published article by Yuan et al., multi-omics detected increased biofilm formation via *Salmonella typhimurium* M3 induced by sub-inhibitory concentrations of tetracycline [87].

By targeting host components involved in the formation of bacterial biofilms and deepening the research on the effects of administering antibiotics (at doses below the minimum effective dose), future studies to characterize new treatments against biofilm-associated bacterial infections may be considerably more relevant and precise.

## 6. Bacteria Detection in Biofilms

In recent years, one of the most challenging problems with biofilm has been the diagnosis of biofilm infections. Mature biofilms tend to resist or tolerate antibiotics and disperse planktonic bacteria into the physiological environment, propagating the infection [88]. In this way, biofilm infections usually reach the chronic phase. Antibiotic treatments sometimes contain but do not eradicate bacteria. Overall, currently, the diagnosis of biofilm infection is based on two principles: clinical evidence and microbiological data [89].

Clinically, biofilm infection might be suspected if a patient presents with any of the following clinical manifestations: (1) patients with or without prosthetic heart valves or pacemakers presenting with intermittent fever and bacteremia with an identical pathogen and without an obvious focus, but higher C-reaction proteins (CRP) and/or erythrocyte sedimentation rates (ESR) with or without leukocytosis (for endocarditis); (2) cystic fibrosis patients with mucoid *P. aeruginosa* in sputum (for *P. aeruginosa* biofilm in cystic fibrosis); (3) patients with a central venous catheter or hemodialysis catheter who have recurrent bacteremia with an identical pathogen (for intravenous catheter (IVC) biofilm); (4) patients with urinary catheters who have recurrent urinary tract infections with the same pathogen (due to urinary catheter biofilm); (5) patients with a joint prosthesis who have chronic pain locally and signs of the loosening of the prosthesis (due to orthopedic infection); and (6) patients with chronic wounds and recurrent wound infections (due to wound biofilm) [89,90,91].

Common microbiological tests include sample collection, microbial culture, and the characterization of antibiotic susceptibility [89]. For example, in individuals with device-associated infections, up to four–five tissue biopsies from various sites close to the device are critical [89]. However, usual microbiological tests are meaningful and useful for the diagnosis of many bacterial infections but are less sensitive in detecting biofilm infections. Therefore, new methods should be introduced to efficiently complement the usual microbiology. It has been demonstrated that the adequate sonication of removed prosthesis could significantly improve the detection rate of bacterial cells [90].

In usual microbiological tests, negative samples from patients with clinical suspicions of biofilm infections are detected. 16S rRNA polymerase chain reaction (PCR)/sequencing [91] as well as the complex and costly shotgun metagenomic sequencing (sNGS) can play a role in the diagnostic evaluation of patients with culture-negative infections [92]. Another technique has also proven useful for recognizing chronic biofilm-associated infection, namely fluorescence nucleic acid in situ hybridization (FISH) [93].

Focusing on orthopedic implant infections, although large international workgroups have recently elaborated consensus criteria to recognize and define them [7], the identification of infections caused by biofilm-forming bacteria or by bacteria able to enter osteoblasts or bone canaliculi remains a thorny problem. And indeed, these infections are extremely insidious, as they are not only able to persist, but also to remain clinically silent or paucisymptomatic for a long time, developing over a period of months to years. Moreover, they tend to slip through classical culture methods. More than 150 years ago, Robert Koch stated the four postulates that gave the theoretical and practical bases on which a causal relationship between a microbe and a disease should be established [94]. Koch’s principles are still used successfully in medical microbiology for diagnosing acute infective diseases caused by planktonic bacteria. But bacteria within biofilms, plunged as they are in a matrix material, the EPS of the biofilm, and most likely adherent to the implant, are prevented from exposing themselves to the culture medium, and this likewise occurs for bacteria shut up in a eukaryotic cell (osteoblast) or nested in the canaliculi. False-negative cultures often lead to the abused diagnostic conclusion of “aseptic failure” even in the presence of manifest clinical signs of infection. 

Sonication, molecular analyses, and advanced imaging techniques have been in turn introduced to improve microbiological diagnosis when conventional cultures fail to point out bacterial contamination despite a well-founded clinical suspicion of infection. And indeed, the sonication of the liquid medium containing the explanted orthopedic prosthesis can help to detach adherent biofilms and disaggregate them, thus releasing biofilm-free bacteria. Detached bacteria can be cultured [90,95,96]. As far as molecular techniques, the already mentioned PCR and FISH seem to improve the detection of bacterial involvement in many cases of persistent infections [97]. Moreover, the multiplex PCR of sonication fluid has been proposed to differentiate between prosthetic joint infection and aseptic failure more accurately [98], while broad-range PCR has been used on biofilms dislodged from knee and hip arthroplasty surfaces using sonication [99]. 

More recently, two sophisticated techniques for molecular analysis, MALDI–TOF MS and PCR-electrospray ionization (ESI)/MS, have been successfully applied to microbiological diagnosis. MALDI–TOF MS (matrix-assisted laser desorption ionization coupled with time-of-flight analysis mass spectrometry) is based on a soft laser ionization of bacteria, or even of the pathological sample, to detect peptide and protein ions of the bacteria cell surfaces through the spectra deriving from their relative masses and charges [100]. The PCR-electrospray ionization (ESI)/MS (Ibis) technique is interestingly founded on nucleotide base ratios (not base sequences) [101]. 

A wider clinical application of next generation sequencing (NGS) could allow the use of this molecular technique in the diagnosis of bone and joint biofilm infections [102]. 

Focusing on imaging techniques, confocal laser scanning microscopy (CLSM), scanning electron microscopy (SEM), and transmission electron microscopy (TEM) can be used advantageously for the morphological study of tissue biopsies and to recognize and characterize bacteria (either embedded within biofilm or intracellularly located) [103,104]. Imaging can help in diagnosing biofilm-associated infections and in addressing the following issues: whether bacteria causative of implant infections enter osteoblasts and whether intracellularly active antibiotics are useful for treating infection.

About the important issue of having a valid biofilm-staining technology that would support surgeons during surgery, there are a series of questions that need to be answered before prefiguring the use of a suitable technique. The limit of detection represents one of the greatest obstacles. And now, excluding the use of radioactive tracers, bioluminescence and fluorescence remain two of the most sensitive techniques in use to reveal in vitro the presence of bacteria and bacterial biofilms. The use of suitable bioluminescent imaging probes has been recently applied for the first time to non-invasive bioluminescence imaging in humans [105]. Appropriate adaptations could probably offer new solutions to achieve a valid biofilm-staining technology. However, much needs to be thought about for the realization of such an approach in biofilm detection, and the clinical application is still far to come. 

The “beacon-based fluorescent in situ hybridization” is another interesting method [106], but is perhaps even more difficult to adapt to the use we would like to make of it. 

A newly developed biofilm-detection method is based on wound blotting on a (nitrocellulose) membrane, then stained with ruthenium red or alcian blue [107]. 

An adaptation of this method to the orthopedic implant infection scenery could guide the surgeon to eliminate the bioburden more precisely. The membrane could be made more adhesive and “attractive” to bacteria with appropriate molecular “baits”. Moreover, this “blotting method” could be improved by using a “reticular grid” to better locate the points in the infected joint to be debrided or cleaned intensively.

Nanotechnologies offer rapidly advancing solutions for the sensitive diagnosis of orthopedic infections [108]. In consideration of the antimicrobial properties exhibited by nanomaterials even on resistant and tolerant bacterial strains that form biofilms [109], of particular interest is the possibility of unifying the ability to diagnose and treat bacterial infections in a single formulation [110,111]. The concept of “nanoteranostics” refers to the strategy of combining, through molecular engineering, imaging probes with therapeutic compounds into a single nanoparticle capable of providing targeted delivery. The great potential but also challenges expressed by nanotheranostics in the detection and treatment of multidrug-resistant and biofilm-forming S. aureus were recently discussed by Mosselhy et al. [112].

## 7. Immunometabolism 

There is a close relationship between the immune system and the host’s metabolic system. The pathogen plays an important role in this relationship. Recent studies have highlighted the continuous crosstalk between bacteria and immune cells. The metabolic interaction between pathogenic (biofilm) bacteria and host immune cells represents the immunometabolism of (biofilm) infection.

The relationship between the host metabolic system and the bacterial counterpart, with its metabolic processes and virulence mechanisms, evolves through infection [113]. Bacterial infection emphasizes the tight association between the host metabolic system and bacteria. 

In terms of bacterial infections, immunometabolic evaluations have focused on the planktonic mode of growth [114]. Given the differences in the inflammatory behavior of immune cells against planktonic versus biofilm-associated infections, studies should also specify the metabolic properties of immune cells in biofilm infections [114]. 

The host immune reaction against biofilm infection is mostly ineffective, which results in chronic infections [88]. 

The anti-inflammatory milieu that develops in implant-associated staphylococcal biofilm infections exhibits an abundant infiltration of myeloid-derived suppressor (MDSC) cells and a high production of the anti-inflammatory cytokine IL-10 [115]. In this connection, it was recently discovered that *S. aureus* can upregulate the expression of genes correlated with a lowering of immune cell defensive functions [116]. 

A key molecule (which we will also recall later in the paragraph on metabolomics) that mediates the process of dampening the immune defenses is the metabolite itaconate, a metabolite produced by the tricarboxylic acid (TCA) cycle in immune cells. Bacteria, once internalized in the immune cells, would switch off the oxidative burst by stimulating neutrophils to produce itaconate [117]. The reprogramming of leukocyte immunometabolism by bacteria seems to be an important and insidious mechanism of the chronicization of biofilm infection.

Metabolic adaptability is an important characteristic of *S. aureus*, as it allows the bacterium to survive even in adverse environmental conditions. Tomlinson et al. have demonstrated that *S. aureus* adapts to itaconate by producing biofilm. Itaconate inhibits glycolysis in *S. aureus*. Itaconate-adapted *S. aureus* strains, as those isolated from chronic infections turned out to be, showed low glycolytic activity, high EPS production, and formed biofilms even before itaconate stimulation [118].

Most of the time, the immune response of the host to a biofilm leads to long-term infections. Numerous routes have been demonstrated to be involved in this phenomenon, including direct interactions with neutrophils, macrophages, and MDSCs [119,120].

The characterization of whether and how biofilm avoids host immune-mediated killing could support advanced therapeutic approaches to enhance the immune responses and facilitate the clearance of biofilm infections. 

However, great attention should be paid when manipulating metabolic reactions in immune cells to develop new therapeutic strategies against biofilm infections. 

Indeed, the perpetuation of inflammation, with the consequent lack of immune resolution, is due to the presence of a biofilm that frustrates phagocytosis. Potentiating proinflammatory responses will not necessarily solve the immune cells’ battle against biofilm, since biofilm itself, with its irremovable persistence, is the cause of the frustration [120].

The competition for oxygen and micronutrients during immune responses can alter the local environment. Cancer cells are voracious of micronutrients and glucose [121] and, as a result, slow the rate of glycolysis in tumor-invading lymphocyte cells [121,122]. Bacteria exhibit similar voracity in biofilm infections. Indeed, microbial infections contend with the host immune system for oxygen, glucose, and micronutrients [123]. Due to the increased oxygen consumption by bacterial and immunological cells during *S. aureus* infection, a regional hypoxia might result [123]. 

Arginase and indolamine-2, 3-dioxygenase are two enzymes released by various cells in the inflammatory sites to consume micronutrients in the peripheral niches [124]. 

A gradient of oxygen concentration is one of the most frequent conditions of biofilm in oxic situations. Respiring bacterial cells use oxygen at the surface layers of the biofilm, causing an oxygen limitation condition that alters the bacterial metabolism and creates anaerobic conditions inside the lower layers of EPS [125].

Hypoxia was detected, for instance, in non-healing wounds infected with obligate anaerobic microbes such as *Clostridia* and *Bacteroides*, which are typically isolated from chronic wounds [126,127]. In this sense, those bacteria that require extremely low oxygen levels for their growth can create anoxic niches in the afflicted areas. 

Oxygen concentration is essential for both neutrophil activity and microbial persistence in biofilm infections. Wu et al. analyzed the role of local oxygen content in the genesis of bacterial biofilm infection. Both bacteria and host cells absorb oxygen, regulate oxygen transport, and react actively to oxygen, this resulting in interactions and competitions [123]. Wu et al. found that in the proximity of a biofilm infection, the co-consumption of oxygen by both host neutrophils and biofilm-embedded bacteria promotes the development of hypoxic conditions. The generation of reactive oxygen species (ROS) requires adequate oxygen levels. If the hypoxic conditions persist, the ability of neutrophils to create ROS (such as hypochlorous acid and hydrogen peroxide) consistently decreases with a consequent reduction in the neutrophil-killing ability [123]. Moreover, the finding that hypoxia can readily establish in the interior of the biofilm is also biologically relevant, because this change will alter microbial metabolism and persistence. 

## 8. Metabolomics of Biofilm as Diagnostic and Therapeutic Target

Because of their poor ability to predict infection in the presence of confounding processes (such as noninfectious inflammation), to predict disease outcomes, and to guide and evaluate treatment regimens, the available biofilm infection biomarkers appear inadequate. For this reason, it is crucial to search for novel and efficient clinical biofilm biomarkers.

Metabolomics enable the comprehensive analysis of small molecules, known as metabolites, which are the end products of cellular processes and reflect the metabolic status and interactions within biofilm communities [128]. Integrating metabolomics with other omics technologies and biofilm research could pave the way for more effective solutions in the field of biofilm science [129]. However, the metabolomic profiling of biofilms is not without challenges. Biofilms exhibit heterogeneity in terms of metabolic activities and composition, which can lead to variations in metabolite profiles within the same biofilm community [130,131]. 

The metabolic responses of bacteria in biofilms are different from those of their planktonic counterparts, according to several works. The presence of distinct glycolytic enzymes between biofilms and planktonic bacteria highlights notable differences in their metabolic processes. Noticeably, the enzyme glyceraldehyde-3-phosphate dehydrogenase (GAPDH) shows significantly higher expression in biofilm forming-strains [130]. 

Moreover, *S. aureus* biofilm formation relies on tricarboxylic acid (TCA) intermediates, while *S. epidermidis* biofilms thrive in nutrient-rich environments, when TCA activity is suppressed [132]. 

A prominent finding is that, in *S. aureus*, the enzyme Glucose 6-Phosphatase (G6P) leads, through a phosphorylation (that of the histidine-containing protein) and subsequent concatenated biochemical steps, to the activation of *CcpA*. *CcpA* (coding for the catabolite control protein A) regulates the transcription of the operon *ica*ADBC, thus favoring the accumulation of PIA [133]. 

By regulating the physical contact between bacterial cells and their immediate surroundings, matrix formation not only configures the biofilm structure but also results in metabolic diversity. This permits the metabolic cross feeding that favors the emergence of metabolically distinct subpopulations inside a biofilm and transforms the latter into a metabolically diverse community [134]. Signals that promote metabolic differentiation are affected by interspecies interactions and biofilm structure, which in turn shape a biofilm’s nutritional and chemical gradient [135]. When one species’ metabolic by-products are utilized as nutrients by another species in a biofilm, interspecies interactions foster metabolic cooperation [134]. The oral biofilm generated by *Streptococcus oralis* and *Veillonella* spp. is an example of the former using the latter’s lactic acid as a carbon source [136]. 

The regulation of multiple bacterial phenotypes depends on various cyclic dinucleotides (c-di-NMPs) that represent an intracellular signaling second-messenger system. An increase in cyclic diguanylate monophosphate (c-di-GMP), the first cyclic dinucleotide identified in 1987 [137], is linked to biofilm development in many bacterial species [138]. With more than 100 c-di-GMP-metabolizing proteins in some species, the c-di-GMP is also the most complex second messenger signaling system discovered in bacteria. This signaling network is especially prominent in the human pathogens *P. aeruginosa*, *Salmonella typhimurium*, *E. coli,* and *Vibrio cholerae*, but is also in the Gram-positive *Clostridium* and *Mycobacterium* species [139]. c-di-GMP controls biofilm development by facilitating cell adhesion to surfaces via a signaling cascade, thereby playing a central role in the switching between the sessile and planktonic modes of bacterial growth [139]. c-di-GMP has a role in the persistence of *P. aeruginosa* biofilms, which are often found in lung infections, since it controls the formation of exopolysaccharide alginate, a significant component of the *P. aeruginosa* biofilm matrix. The rough small colony variations (SCV) of *P. aeruginosa* have an enhanced amount of c-di-GMP and demonstrate greater resistance to antimicrobials [139,140]. 

Second messenger cyclic adenosine monophosphate (c-di-AMP), rather than c-di-GMP, is produced by *S. aureus*, and this results in the production of components, presumably adhesins, needed for biofilm formation [141]. 

A great amount of in vitro evidence has shown that high levels of cyclic dinucleotides govern the initiation of biofilm development by bacteria [142].

**Figure 3:**
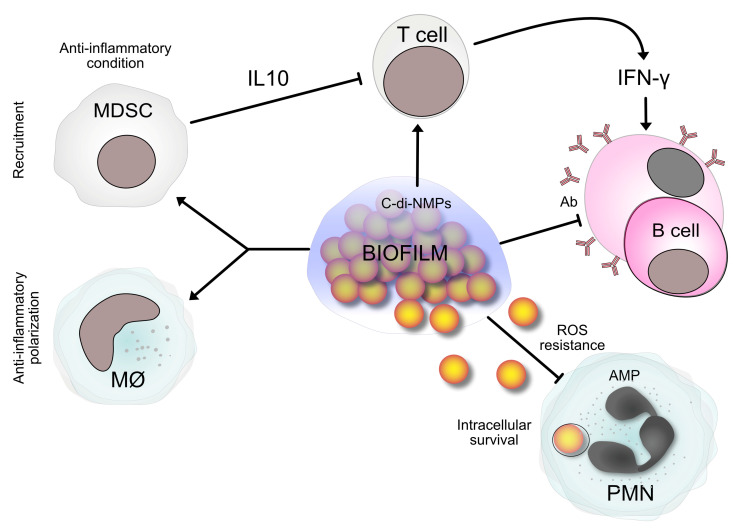
An example of the relationship between immune defenses and bacterial biofilm is summarized, in which c-di-NMPs are involved. Biofilms produce c-di-NMPs. c-di-NMPs stimulate the synthesis of biofilm matrix components. Host cells sense c-di-NMPs as PAMPs. As a result, an immune response is generated to eliminate pathogens. But, the biofilm matrix protects biofilm bacteria as a shield against phagocytosis and antibody deposition by plasma cells. Furthermore, biofilm induces the reprogramming of macrophages towards an M2 phenotype, characterized by poor microbicidal activity, and the recruitment of myeloid-derived suppressor cells (MDSCs) [142]. MDSCs produce the immunosuppressive molecule interleukin-10 (IL-10), which attenuates the immune response and contributes to biofilm persistence. Some bacteria within biofilms can be phagocytosed by immune cells but resist ROS and AMPs (such as defensins 3 and LL-37) thanks to the polysaccharides of their matrix [27,143,144,145]. They can survive intracellularly, thus contributing to the chronic nature of biofilm-associated infections. Abbreviations: c-di-NMPs, cyclic dinucleotides (intracellular signaling second-messenger systems); MØ, macrophages; IFN-γ, interferon-γ; NK, natural killer cells; PAMPs, pathogen-associated molecular patterns; T cells, T lymphocytes; B cells, B lymphocytes/plasma cells (plasma cells derive from B lymphocytes after encountering the antigen); Ab, antibodies; PMNs, polymorphonuclear neutrophils; AMPs, antimicrobial peptides; ROS, reactive oxygen species.

Figure 3 reports a simplified example in which biofilm and c-di-NMPs are involved.

Persister cells are particularly abundant in biofilms. The mechanism of their dormancy is not fully understood but may be due to the expression of toxin–antitoxin systems, which, interestingly, affect the c-di-GMP network and are involved in the regulation of biofilm formation [139]. 

To guide and monitor antimicrobial treatment, as well as to realize biomarkers for biofilm infections, metabolomics is a promising approach, although the context is of great complexity. Biomarkers could be useful to predict infection, causative agents, disease severity, and outcome, and to distinguish infection among confounding clinical outcomes, such as non-infectious inflammatory processes.

Regarding the potential of metabolomics as a basis for anti-infective therapeutic strategies, several studies have put forward proposals. 

Kim et al. studied the effects of the pentose phosphate pathway on the metabolism of *S. aureus*, showing that pentose phosphate pathway mutation significantly impacts ATP levels. Other metabolomics studies have shown that pentose phosphate pathway mutation leads to decreased pyrimidine metabolism, including decreases in ribose-5P, UMP, and GMP. These nucleotide reductions impact the amount of extracellular DNA in biofilms and are associated with reduced both biofilm formation and resistance to oxidative damage [146].

A study by Mao et al. focused on the cell-free supernatant of lactic acid bacteria on *S. aureus* biofilm and its metabolites. The treatment considerably slowed *S. aureus* growth and prevented it from forming a biofilm. Important metabolic pathways such amino acids and carbohydrates metabolism were among the most noticeably altered metabolic pathways [147].

Recently, attention has been paid to itaconic acid, a metabolite generated by the TCA cycle in eukaryotic immune cells, and its synthetic derivative dimethyl itaconate.

This is, counterintuitively, the same metabolite that bacterial-mediated upregulation has been shown to be responsible for switching off the defensive activity of immune cells (see the previous paragraph). As we have previously stated, the interpretation of the different faces of the immune response cannot be black or white: it has many nuances, which makes us consider how much research and how much caution should be spent in designing new treatments that involve the immune system.

The results of some very recent studies follow.

Xie et al. in their new experimental study focused on itaconic acid and dimethyl itaconate, showing for the natural metabolite and its derivative an antibacterial and antibiofilm activity through the TCA cycle in a carbon-enriched environment. 

It has been emphasized that itaconate plays a critical role in linking immune and metabolic functions to influencing host defense [148]. 

Hooftman et al. emphasized that certain metabolites can have double lives as immunomodulators. They suggested that itaconate is a valid example of this concept, having multiple anti-inflammatory effects in macrophages [149]. 

The study by Zhu et al. investigated the mechanism by which itaconic acid functions as an antibacterial metabolite in macrophages. Their experimental results showed that itaconic acid significantly promoted the pentose phosphate pathway, which subsequently led to significantly higher NADPH oxidase activity and more reactive oxygen species production [150].

Kim et al. report host-directed therapeutic effects against mycobacterial infections. In their study, itaconate significantly suppressed the production of interleukin-6 and 10, whereas it enhanced phagosome maturation [151]. 

Riquelme et al. demonstrated that *P. aeruginosa* utilizes host-derived itaconate to redirect its metabolism to promote biofilm formation [152]. They showed that *P. aeruginosa* can exploit the host immune response to maintain infection. They found that *P. aeruginosa* alters its metabolic and immunostimulatory properties in response to itaconate. Itaconate induces bacterial membrane stress, resulting in the downregulation of lipopolysaccharides and the upregulation of exopolysaccharides. This itaconate-adapted *P. aeruginosa* accumulates mutations that favor biofilm formation. Exopolysaccharides, in turn, induce itaconate production via myeloid cells, thus traducing the host immune response to a behavior that encourages chronic infection. 

Indubitably, the bacterial metabolic adaptability (of which *P. aeruginosa* gives us an example) needs to be considered when designing therapies [152]. 

Importantly, some intracellular pathogens have evolved to produce itaconate-degrading enzymes, which are required for intracellular proliferation and to promote pathogenicity. To our reassurance, Hammerer et al. presented a molecule able to re-sensitize *Salmonella enterica* to itaconate [153].

## 9. Conclusions

Biofilm infections involve serious complications and significant economic burdens because they are frequently refractory to antibiotic therapy. Currently, the systemic administration of antibiotics and antibiotic-doped biomaterials are used as a prophylactic and therapeutic measure in patients undergoing implant procedures. However, this approach has some drawbacks, such as the risk that only an inadequate concentration of antibiotic (below the minimum inhibitory dose) reaches the site, stimulating the emergence of bacterial dormancy and antibiotic tolerance. The study of bacterial biofilms, from their role in orthopedic infections to their impact on immunometabolism and metabolomics, represents a various and complex field of research that continues to produce promising insights. However, as we explore the many facets of biofilms, questions continue to arise. The hope is to find innovative strategies in the management of biofilm-related infections and to exploit the immune response to fight them. However, any strategy involving the manipulation of immune pathways must be evaluated with care and caution in view of the potential damage resulting from the induction of persistent inflammation in tissues.

Looking to the future, further investigations into bacterial biofilms should strengthen the interdisciplinary approach, combining microbiology, immunology, and biochemistry to unravel the intricate web of interactions within biofilms. The development of advanced imaging techniques will be crucial in gaining a deeper understanding of biofilm dynamics and host responses. This new knowledge will benefit the search for new weapons to counteract biofilm infections and to mitigate their impact on human health.

Biotechnological advances in fields such as nanotheranostics raise real hope for new successful strategies that combine unprecedented sensitive diagnostic probes with the targeted delivery of powerful antibacterial and antibiofilm molecules in a single multifunctional nanoparticle. In this regard, nanotheranostic platforms exhibit enormous potential for the treatment of both oncological [154] and infectious [112] diseases. Nonetheless, there are still many challenges to face, including very important ones posed by the safety issues associated with the clinical use of nanomaterials.

## Data Availability

Data are contained within the article.
